# Elevating Subjective Well-Being Through Physical Exercises: An Intervention Study

**DOI:** 10.3389/fpsyg.2021.702678

**Published:** 2021-12-17

**Authors:** Katarzyna Iwon, Julia Skibinska, Dorota Jasielska, Sonia Kalwarczyk

**Affiliations:** Institute of Psychology, The Maria Grzegorzewska University, Warsaw, Poland

**Keywords:** physical activity, subjective well-being, happiness, life satisfaction, self-esteem, exercises

## Abstract

**Background**: Physical activity is associated with higher levels of subjective well-being. However, little research has been conducted in naturalistic conditions with a longitudinal design. In the current study, we aimed to examine whether regular activity initiation can impact happiness, life satisfaction, and self-esteem 4 weeks later.

**Methods**: The sample (*N* = 217, 124 women) was divided into three groups based on level of physical activity (active people, beginners, and inactive people). The participants completed measures of happiness, satisfaction with life, self-esteem, and a survey on physical activity. Ninety-five of participants who completed the same set of measures sent by email after 4 weeks were included in the analyses.

**Results**: The study showed a strong relationship between subjective well-being and physical activity. Active people showed higher levels of happiness and self-esteem compared to beginners and inactive people and a higher level of life satisfaction than inactive people. Furthermore, after 4 weeks of exercising, beginners revealed greater life satisfaction and happiness compared to the baseline.

**Conclusion**: These findings confirm that regular physical activity leads to higher levels of well-being. It seems that even a short engagement in physical activity (4 weeks) may contribute to an increase in subjective well-being.

## Introduction

Sport is considered to be the foundation of a healthy life. More and more studies have shown that physical activity is necessary not only for physical (Blair et al., 2004; Penedo and Dahn, 2005; Warburton and Bredin, 2017) but also for mental health (Paluska and Schwenk, 2000; Biddle and Asare, 2011; White et al., 2017). A healthy lifestyle is becoming more and more popular and desirable worldwide, especially among younger generations (Park and Kang, 2008; Cypryańska and Nezlek, 2019). In general, adults present a decreasingly healthy lifestyle over the years (King et al., 2009). However, existing research findings are not conclusive because a healthy lifestyle also plays a significant role among the elderly. A healthy lifestyle is more common among women, more educated people, those who are less focused on sensation seeking, those who have a tendency to plan ahead, and those who perform fewer social roles (Divine and Lepisto, 2005). Proper education enables the development of appropriate habits related to physical activity and medical checkups (Park and Kang, 2008).

A global trend has shown that the rate of insufficient physical activity has remained stable over the past 15 years. Furthermore, physical activity is generally less frequent in highly developed countries than in poorer ones (Guthold et al., 2018). However, in several affluent countries (like England), increased physical activity has been reported before COVID-19 pandemic (Active Lives Adult Survey November 2019/20 Report, 2021). In European Union countries, two out of five residents exercise at least once a week and 7% at least five times a week. The largest proportions of physically active people live in Finland, Sweden, and Denmark. The lowest proportions of physically active people were reported in Bulgaria, Greece, and Portugal. A total of 56% of Poles neither exercise nor do sports (Special Eurobarometer 472 Sport and Physical Activity, 2018; Sport England, 2018). Unfortunately, lockdown led to an even more significant decrease in physical activity (Active Lives Adult Survey November 2019/20 Report, 2021).

Physical activity is associated with increases in many aspects of subjective well-being, such as happiness, life satisfaction, and mood regulation (Paskova, 2010; Maher et al., 2014; Pengpid and Peltzer, 2019; Stolarska et al., 2019; An et al., 2020; Van Woudenberg et al., 2020). A question remains as to the extent to which these beneficial effects are causal and evoked by physical activity or whether other mechanisms are lying under the observed phenomenon. Finding these answers seems to be an important undertaking, especially in the current context, the global COVID-19 pandemic badly affecting both physical and emotional well-being, with people actively searching for strategies that can help them to cope with the emotional consequences of lockdown (Bodecka et al., 2021). In this study, we investigate whether commencing physical activity positively affects happiness, life satisfaction, and self-esteem after 4 weeks of exercising. We also compare the differences among groups presenting various levels of physical activity.

People want to be happy, no matter how they perceive it (Myers and Diener, 1995; Diener and Seligman, 2002; McKee, 2017). Concepts of happiness, subjective well-being, and self-worth have been studied and described by many theorists over the years (Campbell, 1976; Shin and Johnson, 1978; Fordyce, 1988; Diener, 2000; Cast and Burke, 2002; Seligman, 2005, 2018). Researchers use happiness and life satisfaction as indicators of subjective well-being. Nevertheless, the two terms capture different aspects of this phenomenon, which has two dimensions: emotional and cognitive (Diener et al., 1999). The former relates to the experience of a positive emotional state and can be reflected by happiness, whereas the latter represents the global evaluation of one’s life, denoted by one’s satisfaction with life. In the current study, we decided to assess both constructs to obtain a more complex picture of subjective well-being.

There is no consensus regarding the definition of happiness used by philosophers and social researchers. Fordyce (1971) describes happiness as a specific type of emotion, an overall evaluation summarizing all pleasant and unpleasant experiences from the past. According to Diener (2000), happiness indicates how much people like their own lives. In his model of happiness, Seligman (2005) took into account three elements: pleasure, sense of meaning, and commitment. In 2011, Seligman re-examined this model and suggested a new one called PERMA, an acronym for his five measurable well-being components: positive emotion, engagement, relationships, meaning, and accomplishment (Seligman, 2018). Positive emotions include emotions, such as hope, joy, love, compassion, and gratitude, and they can improve level of well-being (Fredrickson, 2001). Engagement is defined as living at the moment and fully focusing on the task (Seligman, 2011). Positive relationships in the model of Seligman (2011) refer to interactions that people have with family, partners, friends, colleagues, and community and to the sense of being valued and loved. Meaning shows individuals that there is something more important than one’s self. What is more, life purpose helps to build a sense of value and worth. Accomplishments (also known as achievements) are considered as strive to the success and mastery and can lead to activation of the other components of PERMA (Seligman, 2011). The PERMA constitutes contribute to subjective well-being but also leads to decreased psychological distress (Forgeard et al., 2011). According to Seligman (2011), each domain can be pursued separately, but relationships between them provide wider spectrum of well-being. A number of researches support this broad understanding of well-being including both hedonic and eudemonic aspects (Huppert and So, 2011; Butler and Kern, 2016; Goodman et al., 2017).

Happiness is also considered the highest good, a positive internal experience, and a definitive incentive for all behaviors (Argyle, 2001). Happiness is also considered as multidimensional construct consisted of cognitive and emotional aspects (Hills and Argyle, 2001). According to the approach proposed by Lyubomirsky (2008), happiness is related to the experience of joy, contentment, and positive well-being, combined with the awareness that life has sense and is both good and meaningful. All these definitions focus on slightly different elements of happiness. In line with the concept of Seligman (2018), we assume that a feeling of happiness is a subjective assessment of the possibility of experiencing happiness in life by referring to such spheres as accomplishments, positive relationships with other people and the world, life engagement, finding a purpose, and feeling positive emotions.

Life satisfaction can be considered a cognitive aspect of subjective well-being (Diener et al., 1985; Pavot and Diener, 1993). Shin and Johnson (1978) define it as a general assessment of quality of life, related to personal criteria. Thus, one’s evaluation of life satisfaction results from a comparison of one’s situation with its established standards. An alternative definition (Gotay et al., 1992) describes life satisfaction as consisting of two elements: ability to cope with daily tasks and one’s satisfaction with functioning in all life areas. Anand (2016) claims that life satisfaction is the best measure of how people feel about their lives. Given that the two concepts of happiness and life satisfaction complement each other, we assumed that it would be beneficial to assess both of them in the current study.

Self-esteem is also related to the concept of well-being and is sometimes included as an indicator of it (Ryff and Keyes, 1995; Cypryańska and Nezlek, 2019). Self-esteem is considered an evaluative aspect of self-concept, which refers to the self or specific fields of the self, like physical attractiveness, social standing, career path, or school achievements (Michalos, 2014). According to Leary and Baumeister (2000), it is the inner psychological monitor of social belongingness, representing a general, subjective view of the self as either worthy or unworthy. Self-esteem has been repeatedly regarded as an evaluation or an attitude. Among others, Rosenberg (1965) has stated that self-esteem refers to an overall assessment of one’s value, which may be represented by an attitude toward oneself, especially one’s own abilities or other socially important features. Depending on one’s subjective evaluation and emotional attitude toward one’s own characteristics, self-esteem can be either positive or negative. Strelau (2000, p. 573) presents a different definition of this concept. He believes that self-esteem is an evaluation of the self, understood as a generalized, relatively persistent assessment of oneself as a person. Harter (2012) also defines self-esteem as a stable trait that is built during one’s life. As components, it includes a rating of physical attractiveness, interpersonal skills, sense of humor, and various other skills. Numerous studies indicate a positive relationship between self-esteem and happiness among children (Dai and Chu, 2018) and adults (Tan et al., 2017).

Although the relationship between physical activity and subjective well-being has been studied more than once, researchers are still finding new fields to explore. Physical activity is defined as muscle work characterized by over-resting energy expenditure (Caspersen et al., 1985). It can affect the life quality of people of all ages (Rottermund et al., 2015; Cihan et al., 2018; Gümüş and Isik, 2018). Comparative studies conducted in clinical groups indicate that sport practiced regularly improves people’s moods and prevents depression (Blumenthal et al., 1999). One study has indicated a significantly higher sense of life quality, life satisfaction, and self-esteem among dance class participants (Gałuszka, 2017). Even a small amount of physical activity increases adolescents’ subjective well-being and self-satisfaction and contributes to more frequent experiences of positive emotions (Paskova, 2010). Those effects are often explained by the secretion of endorphins, which increase during exercises and improve mood (Starosta, 1995).

A higher level of happiness or life satisfaction is one of the many significant benefits of doing sports. Engaging in physical activity or participating in sports is associated with improvements in life satisfaction and self-assessed health status (Zullig and White, 2011; Orlowski and Wicker, 2017; Schmiedeberg and Schröder, 2017). Sport is also considered a beneficial way to spend free time (Richards et al., 2003; Lorenzo et al., 2018). Researchers have noticed a positive effect between active rest and leisure satisfaction among male students. However, for both sexes, leisure satisfaction is associated with improved well-being, understood as increased life satisfaction and decreased stress (Shin and You, 2013). A sense of happiness is more closely related to membership in a sports organization than other recreational activities (Balish et al., 2016). The beneficial effect of physical activity has been observed for different types of sports. For example, in one study the successful completion of a 45-min aerobic session was found to lead to mood enhancement in female students (Stolarska et al., 2019). In another investigation, people practicing recreational tennis manifested a higher level of happiness than those who spent their free time passively (Majewska, 2011). Moreover, Cypryańska and Nezlek (2019) found that different aspects of well-being such as self-esteem, self-efficacy, life satisfaction, and positive effect increased when participants were running in organized races.

Regular exercises affect various aspects of well-being beyond improving mental health. According to Franks and Howley (1998), physical fitness is strongly associated with health and can lead to optimal life quality. When dosed properly, it affects both the physical and the mental self (Carr, 2009). People engaged in regular physical activity tend to have higher levels of self-esteem, optimism, and happiness than inactive physical adults (Cekin, 2015). Self-esteem also increases regardless of the practitioner’s age. Physical activity is associated with increased self-awareness and self-esteem among children and adolescents (Liu et al., 2015). The situation seems to be similar in the case of older people. Indeed, the physical activity of older people brings higher levels of self-esteem and is associated with better social status and relationships (Algarín and Sarasola-Sánchez-Serrano, 2018). Another study has found that physically active women experience more positive feelings and emotions and less sadness, dissatisfaction, senselessness, loneliness, exhaustion, and harm (Demuth and Czerniak, 2011). Sport, combined with a properly balanced diet, is a good premise for improving life quality and increasing self-esteem (Zurita Ortega et al., 2018). Sports and diets are directly related to self-esteem, perceived physical fitness, and body image (Zamani Sani et al., 2016).

Although the beneficial effect of physical activity on well-being seems to be well established, several issues need to be addressed. First of all, given that the majority of research is correlational, there is little evidence of causal relationships between exercises and happiness from longitudinal studies. Does regular activity lead to elevated well-being? Existing data often comprise clinical samples (Blumenthal et al., 1999; Cooney et al., 2013), and their effects cannot be generalized to healthy populations. To address the issues mentioned above, we designed a study to further explore the links between subjective well-being and physical activity in a naturalistic setting. Conducting a study in a naturalistic design supports the practical relevance of the research for designing future interventions that can elevate various aspects of subjective well-being.

In the current study, we compared the levels of happiness, satisfaction with life, and self-esteem among three groups based on their level of physical activity: participants who exercise regularly in a fitness club or a similar facility (Group 1 – active people); people who were just starting to engage in physical activity (Group 2 – beginners); and those who do not exercise (Group 3 – inactive people). Furthermore, to investigate the impact of exercise on well-being, we conducted our measures twice: at the moment of initiation for Group 2 and 4 weeks later. Based on the studies described above that indicate the existence of a relationship between physical activity and a sense of happiness and life satisfaction (Paskova, 2010; Zullig and White, 2011; Nowak, 2013; Siwy-Hudowska, 2013; Schmiedeberg and Schröder, 2017; Zayed et al., 2018), we expected that people who exercise regularly would show a higher level of happiness (H1) and life satisfaction (H2) than beginners in physical activity and people who do not practice sport. Based on the results of the previous research showing a positive relationship between physical activity and positive self-esteem (Moral-García et al., 2018), we expected that active people would have higher self-esteem than the other two groups (H3). According to the PERMA concept, physical activity should increase well-being because it is connected to all of the components:

- P (positive emotions): Endorphins are released during sport activities (Starosta, 1995),
- E (engagement): Sport may become a passion which gives individuals state of flow (Puig Barata, 1995),
- R (relationships): Regular physical activity at the gym or in the group impacts sense of belonging and helps building relationships with people with similar interests (MacPhail et al., 2004),
- M (meaning): Physical activity helps people to take care of their health, so it can be considered as conscious self-care and chance to self-realization (Khan et al., 2012),
- A (accomplishments): When practicing sports, people can easily observe positive effects such as increase in the level of energy and fitter figure (Chaput et al., 2011).

To assess whether the obtained effect is persistent and exercising leads to changes in well-being, we decided to re-measure all the variables after 4 weeks. We were encouraged by the results of Wicker et al. (2015) demonstrating that life satisfaction increased after participating in four-week fitness programs. In this study, we expected changes not only in the level of life satisfaction but also in happiness and self-esteem in the group of beginners (H4). Therefore, our study represented an extension of the research conducted to date. Investigating these relationships was a noteworthy undertaking as the results could help design future interventions aimed at increasing subjective well-being.

## Materials and Methods

### Procedure and Participants

All participants were volunteers. They were asked in person or indirectly by research assistants to complete self-report questionnaires. Regular exercisers were recruited in facilities belonging to a fitness club chain in Warsaw and the surrounding area. Clients of those facilities were approached at the reception desk and proposed to participate in a study on the relationship between physical activity and well-being. Approximately 30% of participants approached in the gym agreed to participate in the study. Beginners and inactive people were recruited among residents of Warsaw and its surroundings. The respondents were scouted among researchers’ friends through social media and in location such as universities and workplaces. The snowball effect was also used in the study. Some beginners were new clients of fitness clubs and gyms.

The study lasted over 2 months, from January 11 to March 19, 2019. Initially, the respondents received a set of paper questionnaires (first survey) with a separate card to write down their email address and date of entering the study. Four weeks after completing the first survey, participants received at the provided email address a link to an online survey (second survey). In addition, two emails were sent with a reminder to complete the questionnaires 2 days apart. Informed consent was obtained from all participants, and all were informed of their responses’ anonymity. There were no incentives for participation. All procedures performed in the study were in accordance with the ethical standards of the relevant institutional review board and with the 1964 Declaration of Helsinki and its later amendments or comparable ethical standards.

The sample consisted of 295 people, although 78 were later excluded either because: (1) people under the age of 18; (2) people who declared exercising once or twice a week (we decided to exclude this group in order to compare active persons with inactive ones); and (3) people who exercised before the study but did not plan to exercise in the following month at the gym or fitness club because they did not meet our criteria and did not qualify for any of our groups. Further statistics included 217 participants. A total of 57.1% of the respondents were women, 41.9% were men, and 1% were people who did not identify with either of these genders or did not answer the question. Women accounted for 54.1% of the active people, 56.7% of beginners, and 60.5% of inactive people. The respondents’ ages ranged from 18 to 64 years (*M* = 29.57, *SD* = 9.11). The average age in the studied groups was as follows – active people (*M* = 30.51, *SD* = 9.40), beginners (*M* = 26.97, *SD* = 6.59), and inactive people (*M* = 30.95, *SD* = 10.30). The majority of respondents (55.3%) declared completing higher education, followed by secondary education (37.8%), vocational education (4.7%), and primary education (1.4%). Two people did not answer the question about education. Most of the people who exercised had higher education 55.4%, followed by secondary education 37.8%. Among beginners, 67.2% had higher education and 31.3% secondary education. Among those who do not exercise 44.7% declared higher education, 43.4% secondary education and 9.2% vocational education. Table 1 shows the age, gender, and education data divided by groups for both measures. Most of the participants were employed (56.2%). Studying and working respondents accounted for 24.9% of the sample and just studying 14.7%. The least numerous group (4.1%) represented unemployed people who were not studying.

**Table 1:** Demographic data (age, gender, and education level) data divided into groups for first (T1) and second (T2) measurement.

		*N*	Age	Gender		Level of education	
T1	Active people	74	30.51	Female	54.1%	Primary education	0%
				Male	44.6%	Vocational education	4.1%
				No data	1.4%	Secondary education	37.8%
						Higher education	55.4%
	Beginners	67	26.97	Female	56.7%	Primary education	1.5%
				Male	41.8%	Vocational education	0%
				Other	1.5%	Secondary education	31.3%
						Higher education	67.2%
	Inactive people	76	30.95	Female	60.5%	Primary education	2.6%
				Male	39.5%	Vocational education	9.2%
						Secondary education	43.4%
						Higher education	44.7%
T2	Active people	23	30.57	Female	65.2%	Primary education	0%
				Male	34.8%	Vocational education	8.7%
						Secondary education	43.5%
						Higher education	47.8%
	Beginners	34	28.71	Female	61.8%	Primary education	0%
				Male	35.3%	Vocational education	0%
				Other	2.9%	Secondary education	32.4%
						Higher education	67.6%
	Inactive people	38	29.39	Female	65.8%	Primary education	2.6%
				Male	34.2%	Vocational education	5.3%
						Secondary education	42.1%
						Higher education	50.0%

The participants were divided into three groups based on their frequency of physical activity: active people (74 participants), beginners (67 participants), and inactive people (76 participants). Based on the Chi-square test, it can be stated that the groups were equinumerous, so the distribution was not significantly different from the random distribution, *X*^2^(2, *N* = 217) = 0.62, *p* = 0.734. Physical activity was defined as participation in classes organized by fitness clubs or gyms, requiring physical fitness and endurance and/or performing physical exercises outside fitness clubs or gyms, other than work or housekeeping duties. Active people were considered those who had been doing physical activity for at least 3 months, on average, three times a week or more, for a minimum of 30 min in one exercise session. Beginners were people who had not been doing physical activity or had been doing it irregularly in the past 3 months (on average less than once a week) and expressed a willingness to start physical activity in a fitness club or gym at least twice a week for a month.^1^ Inactive people had not been engaging in any physical activity or had been doing so less than once a week during the previous 3 months and did not express a desire to begin physical activity within the next month.

Only 140 participants completed the second questionnaire. The analysis included 95 people consisting of adult participants and those who met the conditions for being either active, a beginner or inactive. In order to verify Hypothesis 4, the sample was divided into the previously mentioned groups of active people (23 participants), beginners (34 participants), and inactive people (38 participants), according to the actual physical activity undertaken by the participants in the past 4 weeks. The group of active people consisted of people who were physically active at least three times a week during the 3 months before and during the study. The group of beginners consisted of people who exercised at least twice a week during the research yet had not participated in physical activity or had exercised less than once a week during the 3 months before starting the study. The inactive group consisted of people who had not exercised for the previous 4 months. According to the Chi-square test, these groups were also equinumerous: *X*^2^(2, *N* = 95) = 3.81, *p* = 0.149.

### Measures

#### Happiness

The Oxford Happiness Questionnaire (OHQ; Hills and Argyle, 2002; Polish adaptation: Poprawa, 2008) is a measure of human happiness potential, meaning the possibility of experiencing happiness in life. It consists of 29 items (such as “I have very warm feelings towards almost everyone”) and contains a six-point scale of answers ranging from 1 – “I strongly disagree,” to 6 – “I strongly agree.” The reliability of the scale was good: *α* = 0.921 for the first measurement and *α* = 0.943 for the second.

#### Life Satisfaction

We used the Satisfaction With Life Scale (SWLS; Diener et al., 1985; Polish translation: Jankowski, 2015), which assesses a person’s general sense of satisfaction with their quality of life. It contains five statements (such as “I am satisfied with my life”). Participants answer using a seven-point Likert scale (from 1 – “Definitely disagree,” to 7 – “Definitely agree”). The reliability of the scale was good: *α* = 0.830 for the first measurement and *α* = 0.894 for the second.

#### Self-Esteem

We used the Rosenberg Self-Esteem Scale (RSE; Rosenberg, 1965; Polish adaptation: Łaguna et al., 2007), which evaluates a person’s level of general self-esteem, defined as a relatively constant disposition denoting a conscious attitude toward the self, which affects the individual’s emotions, thoughts, and behaviors. It consists of 10 statements (such as “I feel that I have a number of good qualities”). Participants answer using a four-point scale of answers (from 1 – “Strongly agree,” to 4 – “Strongly disagree”). The reliability of the scale was good: *α* = 0.889 for the first measurement and *α* = 0.913 for the second.

#### Survey Regarding Physical Activity

The respondents also completed a survey on physical activity, in which they answered the following closed-ended questions: “How often in the past 3 months have you practiced sports or taken up physical activity?,” “Do you intend to undertake or continue engaging in physical activity at a gym or fitness club in the next month, at least twice a week?,” and “How do you evaluate the level of your activity?” A further question was open-ended: “How long does your average training last? Please enter the approximate time in minutes.” The participants also filled in demographic data, such as age, gender, education, place of residence, and professional situation.

## Results

### Main Results

The analysis began by determining the Spearman’s correlation of dependent variables, taking into account the sample of people over 18 years of age from divided into three groups according to their frequency of physical exercise (*N* = 217). In the first measurement (T1), happiness was found to strongly correlate with life satisfaction (*r* = 0.71, *p* < 0.001) and self-esteem (*r* = 0.74, *p* < 0.001). Life satisfaction was associated with self-esteem (*r* = 0.59, *p* < 0.001). The second measurement (T2) on a sample of 95 people showed stronger relationships between the studied variables. Happiness was found to correlate with life satisfaction (*r* = 0.76, *p* < 0.001) and self-esteem (*r* = 0.78, *p* < 0.001). Greater happiness was associated with higher life satisfaction and self-esteem. Satisfaction with life also correlated with self-esteem (*r* = 0.74, *p* < 0.001). An increase in self-esteem accompanied the increase in life satisfaction.

**Figure 1:**
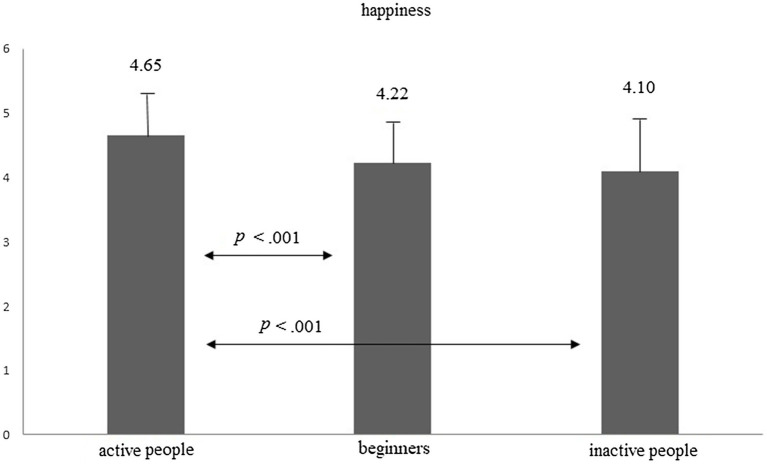
Average level of happiness in groups of active people, beginners, and inactive people.

In order to compare the level of happiness, a one-way ANOVA was performed. The analysis showed that in the first measurement the groups significantly differed in their levels of happiness: *F*(2, 214) = 15.59, *p* < 0.001, *eta* = 0.127. The data are presented in Figure 1. Bonferroni’s *post hoc* tests indicated that active people presented a significantly higher happiness level than beginners and inactive people (*p* < 0.001). Hypothesis 1 was thus confirmed.

**Figure 2:**
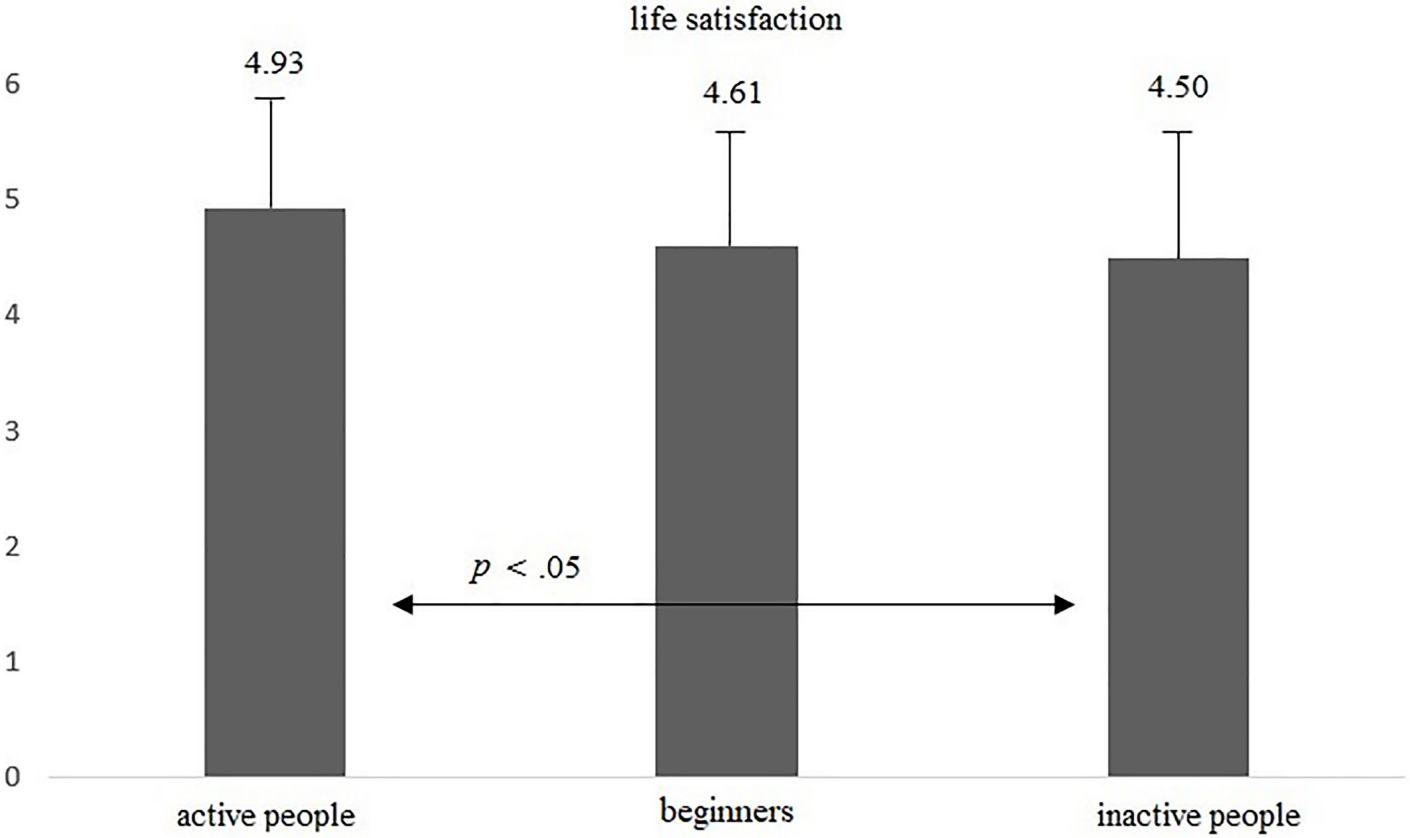
Average level of life satisfaction in groups of active people, beginners, and inactive people.

Consistent with the expectations of Hypothesis 2, the groups at the first measurement also differed in their levels of life satisfaction: *F*(2, 214) = 3.52, *p* = 0.031, *eta* = 0.032. Active people presented a significantly higher level of life satisfaction than inactive people (Bonferroni’s *post hoc* test, *p* < 0.05). The data are presented in Figure 2.

**Figure 3:**
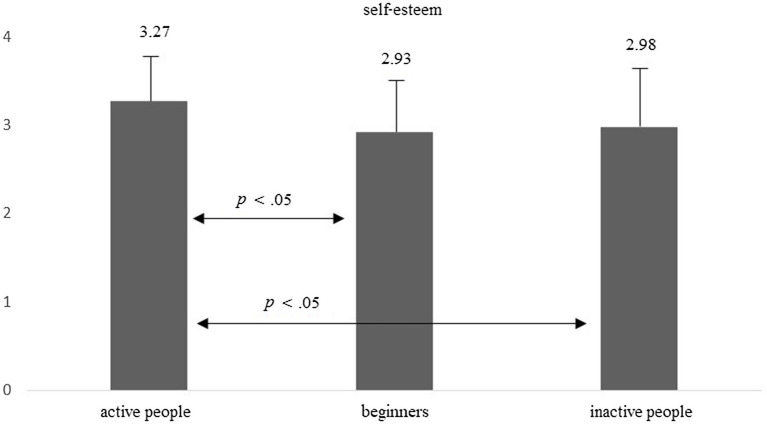
Average level of self-esteem in groups of active people, beginners, and inactive people.

Differences were also observed for the level of self-esteem: *F*(2, 214) = 7.36, *p* = 0.001, *eta* = 0.064. Active people presented a significantly higher level of self-esteem at the first measurement than beginners and inactive people (Bonferroni’s *post hoc* test, *p* < 0.05). The data are presented in Figure 3. Thus, Hypothesis 3 was confirmed.

A Student’s *t* test for dependent samples was used to verify whether commencing exercise leads to a higher level of happiness (H4). As Table 2 shows, after 4 weeks of physical activity, the beginners’ average levels of life satisfaction and sense of happiness increased above the baseline (*p* < 0.05). The results for self-esteem were not statistically significant. The levels of the above variables were also compared in groups of active and inactive people, but after 4 weeks, no significant changes were observed in life satisfaction, sense of happiness, or self-esteem. This was consistent with our expectations, as there was no change in physical activity in these groups.

**Table 2:** Comparison of life satisfaction, happiness, and self-esteem in the group of beginners before and after 4 weeks of physical activity.

	*M*	*SD*	*t*	*p*	*d*
Life satisfaction T1	4.80	0.91	2.19	0.036	0.38
Life satisfaction T2	5.12	0.94			
Happiness T1	4.32	0.65	2.60	0.014	0.45
Happiness T2	4.54	0.67			
Self-esteem T1	2.95	0.60	1.51	0.141	-
Self-esteem T2	3.10	0.44			

Single-factor repeated-measures ANOVA was also performed. The simple effect of time (T1, T2) on the level of life satisfaction turned out to be statistically significant *F*(1, 92) = 7.29, *p* = 0.008. The effect of the interaction of the group (active people/beginners/inactive people) and time on the level of life satisfaction was statistically insignificant *F*(2, 92) = 0.77, *p* = 0.465. The simple effect of time (T1, T2) on the sense of happiness turned out to be statistically insignificant *F*(1, 92) = 0.97, *p* = 0.327. The effect of the interaction of the group (active people/beginners/inactive people) and time on the level of happiness is statistically significant *F*(2, 92) = 5.64, *p* = 0.005. To determine the essence of this effect, a Bonferroni’s *post hoc* test was carried out, which showed that the change in the level of happiness in beginners, *M_1_* = 4.32, *SD_1_* = 0.65, *M_2_* = 4.54, *SD_2_* = 0.67, is greater than in the case of inactive people, *M_1_* = 3.90, *SD_1_* = 0.72, *M_2_* = 3.90, *SD_2_* = 0.82. The simple effect of time (T1, T2) on the level of self-esteem was statistically insignificant *F*(1, 92) = 1.71, *p* = 0.194. The effect of the interaction of the group (active people/beginners/inactive people) and time on the level of self-esteem turned out to be statistically insignificant *F*(2, 92) = 0.81, *p* = 0.447.

Additionally, it was checked whether the participants who completed the second measurement (separate active people, beginners, and inactive people) vary in the level of the studied variables at the first measurement. Differences between the groups were noted. Table 3 shows the data.

**Table 3:** Comparison of life satisfaction, happiness, and self-esteem in the participants who completed second measurement separately for the group of active, beginners, and inactive people.

	*N*	*F*	*df1*	*df2*	*p*	*eta*
Life satisfaction	95	4.65	2	92	0.012	0.09
Happiness	95	11.50	2	92	<0.001	0.20
Self-esteem	95	3.20	2	92	0.045	0.07

Bonferroni’s *post hoc* tests indicated that inactive people presented a significantly lower happiness level, *M* = 3.90 than active people, *M* = 4.73, *p* < 0.001, and beginners *M* = 4.32, *p* < 0.05. Active people presented a significantly higher level of life satisfaction, *M* = 5.08 than inactive people *M* = 4.26, *p* < 0.05. Active people presented a significantly higher level of self-esteem, *M* = 3.26 than inactive people *M* = 2.84, *p* < 0.05.

### Additional Results

Age and sex were not related to the levels of self-esteem, happiness, and life satisfaction in all of the studied groups. There were some small correlations when the education level of participants was considered. The level of education correlated with happiness in the group of the beginners (*r_s_* = 0.31, *p* = 0.011). The higher the level of education of beginners, the higher the level of happiness. A positive correlation was also found between self-esteem and level of education (*r_s_* = 0.34, *p* = 0.004) in the group of active people. The higher their level of education, the higher the level of self-esteem.

The relationships between the psychological variables and self-reported activity were also investigated (T1). As Table 4 shows, positive relationships were noticed between the subjective assessment of activity (from 1 – “I choose to avoid activity,” to 4 – “I am a very active person”) and life satisfaction, happiness, and self-esteem. People who regarded themselves as more physically active were found to be happier, more satisfied with their lives and enjoying higher self-esteem, but the relationships between the variables were weak. Moreover, the frequency of physical activity (from 1 – “Less than once a week or not at all,” to 5 – “Four times a week or more”) was positively associated with life satisfaction, sense of happiness, and self-esteem. The higher the frequency of exercise, the higher the rates of the studied variables. A positive correlation was also noticed between average training duration and sense of happiness and self-esteem. More extended average training was found to be accompanied by an increase in declared levels of happiness and self-esteem.

**Table 4:** Correlations between the happiness, life satisfaction, self-esteem, and physical activity variables.

	Happiness	Life satisfaction	Self-esteem
The frequency of physical activity	0.33^**^	0.15^*^	0.26^**^
Duration of average training	0.26^**^	0.08	0.16^*^
Subjective assessment of activity	0.39^**^	0.22^**^	0.28^**^

** *p* < 0.001;

* *p* < 0.05.

Gender comparative analyses were also carried out (T1). A Student’s *t* test for independent samples showed that women and men significantly differed in self-esteem: *t*(213) = 2.66, *p* = 0.008, *d* = 0.37. Men showed higher self-esteem (*M* = 3.18, *SD* = 0.51) than women (*M* = 2.97, *SD* = 0.63). The ANOVA with group and gender factors showed the main effect of gender in explaining self-esteem: *F*(1, 209) = 6.52, *p* = 0.011. Gender explained 3% of the variability in self-esteem. There were no significant differences between the genders in terms of happiness and life satisfaction.

## Discussion

The relationship between physical exercise and happiness has received considerable attention from researchers. However, research on certain forms of exercise (such as fitness) as well as on the impacts of exercising on well-being is quite limited (Stolarska et al., 2019). In our study, we sought to fill this gap. Therefore, our research was more than a comparative analysis of active and inactive people (Gałuszka, 2017). It was also a longitudinal assessment of the results of exercising for people starting physical activity. To our knowledge, there has been no previous longitudinal research on this matter in Poland. The participants were divided into three groups to ensure a broad spectrum. The study compared various indicators of well-being, such as sense of happiness, life satisfaction, and self-esteem, both among and within participants. It is worth studying psychological constructs under different conditions, in different populations, and at different times, because it allows to state to what extent the observed relationships are culturally universal.

Based on the obtained results, the assumed hypotheses were mostly confirmed. Regular physical activity was found to be associated with higher levels of happiness, self-esteem, and satisfaction with life. Hypothesis 1 assumed that active people would declare a higher level of happiness than beginners and inactive people. The executed analysis confirmed this hypothesis. This result is consistent with previously conducted research (Demuth and Czerniak, 2011; Majewska, 2011; Moljord et al., 2011; Richards et al., 2015; Lathia et al., 2017). Higher happiness in people who exercise regularly may result from their greater level of endorphins, triggering positive emotions, which are components of happiness (Starosta, 1995; Dsouza et al., 2020). People’s change in mood may be affected by biological factors. The appetitive system is associated with reward-seeking behavior, a desire to satisfy bodily needs and sensory pleasure. This brain system is dependent on the dopamine system connected with pleasure. This relationship has been confirmed in studies conducted on rats, which have shown that treadmill running increases dopaminergic activity (Hattori et al., 1993, 1994).

Hypothesis 2 regarding life satisfaction in the examined groups was partially confirmed. Active people showed a higher level of life satisfaction than those who did not exercise. The obtained effects were similar to previous studies, which have clearly shown that people who engage in regular physical activity experience better moods and are more satisfied with their lives than people who do not participate in physical activity regardless of age (Elavsky et al., 2005; Zullig and White, 2011; Martin-Albo et al., 2012; Brodáni et al., 2015; Zayed et al., 2018). However, the difference was not significant between active people and beginners. The beginning of physical activity is associated with reaching a goal, which reduces the divergence between an individual’s current situation and internalized standards (Diener et al., 1985). This may cause the higher evaluation of life satisfaction among beginners. Furthermore, people who are commencing physical activity may have a positive life balance considering their past lifestyle and new active beginning. The lack of difference in levels of life satisfaction between active people and beginners can also be associated with their internal motivation, especially in men (Walczak and Tomczak, 2011; Dodge et al., 2021). People who start regular physical activity may have a positive attitude toward their goals and thus may be as satisfied with life as people who exercise regularly.

The study results also confirmed Hypothesis 3, assuming that self-esteem is associated with the undertaking of physical activity. As expected, the people who regularly exercised showed higher self-esteem than beginners and inactive people. Previous studies have also confirmed this relationship (Elavsky et al., 2005; Cekin, 2015; Gałuszka, 2017; Moral-García et al., 2018). These studies suggest that people who exercise regularly show greater self-esteem and are more optimistic and positive about life than inactive people. It is widely acknowledged that regular physical activity helps with body shaping. Furthermore, self-esteem is related to body perception: People who are dissatisfied with their body tend to declare a lower level of self-esteem (Brytek-Matera, 2010; Asimakopoulou et al., 2020; Watt and Konnert, 2020). Therefore, active people may have a more positive image of their body and thus better self-esteem.

A novel aspect of this research is that it was a longitudinal study comparing subjective well-being before and after 4 weeks of physical activity. To the best of our knowledge, no similar studies have been performed in Poland to date. Hypothesis 4, assuming increased levels of happiness, life satisfaction, and self-esteem in people starting physical activity within 4 weeks, was mostly confirmed. The average intensity of life satisfaction and sense of happiness after 4 weeks of exercise became higher than the baseline. The study of Wicker et al. (2015) showed a similar effect on life satisfaction after a 4-week fitness program. Our study also revealed an increase in self-esteem after 4 weeks in the beginners’ group, but the result was not statistically significant. This may have been due to the brief period of exercising. Four weeks is a relatively short amount of time to notice significant effects of physical activity, including improved appearance, which is directly related to self-perception and self-esteem (Brytek-Matera, 2010; Pop, 2016). After such a period, people may not be able to assess positive changes in their physical attractiveness. Beginners may also not notice improvements in their sports skills and capabilities, which are components of self-esteem according to Harter (2012).

This study also revealed other interesting correlates of physical activity. Even though these dependencies were not initially included in the hypotheses or research questions, the results may be considered an additional source of knowledge and enhancement of this research.

A comparative analysis of gender showed that women and men differed in their levels of self-esteem. Men showed higher levels of self-esteem than women. There were no significant differences between the sexes in their senses of happiness and life satisfaction. Other studies have additionally confirmed that there are no gender differences in these variables. Men have only slightly higher overall self-esteem compared to women. However, there are differences in specific kinds of self-esteem. Higher sport self-esteem characterizes men. Men also tend to be more satisfied with their bodies and physical attractiveness, which may not be consistent with external observers’ opinions (Wojciszke, 2012; Magee and Upenieks, 2019).

The study also revealed positive correlations between frequency of physical activity and senses of happiness, life satisfaction, and self-esteem. In addition, the duration of average training was found to be related to happiness and self-esteem: The longer the training duration, the higher the intensity of the studied variables. These results strengthen the above-mentioned findings of the links between physical activity and subjective well-being. A positive relationship was also demonstrated between the subjective assessment of activity and the measured variables. This may suggest that not only activity understood as physical exercise but also everyday living activity may affect subjective well-being.

This study also had certain limitations. One of the main difficulties of the research was the inability to control the participants’ physical activity. It was not possible to verify whether the respondents’ declarations were true or false. Due to their strong need for social approval, the participants could have declared more frequent physical activity than was actually the case. Due to the naturalistic settings, another challenge was related to the lack of possibility of ensuring equal conditions for all participants. The measures for two groups were carried out in gyms, where people often hurry to fixed classes. Individuals may also choose different kinds of activities. The sample consisted of volunteers, so it is possible that they had different levels of well-being than random sample (Rosenthal and Rosnow, 1975). In future studies, it is advisable to control the activity type to ensure that there are no differences between the specific exercises. Moreover, a group of active people was recruited in gyms. In sports facilities, people are exposed to more frequent interactions with people with similar interests and hobbies, so they have a greater chance of developing interpersonal relationships. Social capital is essential for an individual to achieve happiness (Oshio, 2017). Therefore, it is important to have a sense of belonging to a specific group and to establish positive and satisfying relationships with others (Ciupińska, 2005; Fang et al., 2017). Accordingly, it is worth considering that variables such as other people’s presence and satisfying the need for belonging may have represented mediators in this study. What is more depression symptoms may be a barrier to enhance physical activity, so it is worth to check whether low scores on well-being are connected to symptoms of depression (Achttien et al., 2019). Studies indicate that physical activity and exercise can contribute to better well-being and be a beneficial in the treatment of depression (Ströhle, 2008), but influences may be bidirectional and low subjective well-being may contribute to small activity or even inactivity.

Future research on this subject can benefit from this study and take into account the above limitations. Actual control of physical activity may be hard to implement. Therefore, it would be worth conducting a 4-week intervention involving participants of organized groups exercising at least twice a week. Furthermore, future research could also include additional variables, such as a sense of effectiveness or the main kind of motivation to begin exercises. Four weeks may be insufficient to notice positive changes in appearance and thus enhanced self-esteem, so further studies should consider an extended period of physical activity, like 3 months.

Future research could also look into the role of physical activity in clinical populations because it plays such an important role in promoting subjective well-being (Nunan et al., 2013). Specific clinical populations, such as those with obesity (Kim et al., 2017) or chronic pain (Dysvik et al., 2010; Geneen et al., 2017; Law and Sluka, 2017), may benefit from regular physical activity. This line of research is intriguing because it appears that people with specific health conditions have movement beliefs (Varallo et al., 2020, 2021a,b) that may limit their willingness to engage in physical activity. We recommend to verify whether exposing people with these conditions to a physical activity intervention can change their beliefs about movement and improve their subjective well-being.

Our study has broadened previous findings of the links between subjective well-being and physical activity by presenting data from a naturalistic setting in a longitudinal study on the impacts of physical activity. The results seem to be relevant in the modern world, especially in the face of the COVID-19 pandemic and the closure of gyms and other sports facilities by many governments worldwide. Most of our hypotheses were confirmed, indicating that exercising is an essential factor contributing to various aspects of well-being. Given that a healthy lifestyle and physical activity have become increasingly popular subjects in contemporary society, these data may promote an active lifestyle and frequent physical exercise. These results seem to be particularly relevant in the context of the challenges posed by the coronavirus pandemic, where evidence-based practices aimed at improving well-being are able to provide real support that psychologists can offer to enable people to stay happy and satisfied.

## Data Availability Statement

The raw data supporting the conclusions of this article will be made available by the authors, without undue reservation.

## Ethics Statement

The studies involving human participants were reviewed and approved by The Maria Grzegorzewska University. The patients/participants provided their written informed consent to participate in this study.

## Author Contributions

KI and JS developed the study concept, performed data analysis, and described the results. KI and DJ drafted and revised the manuscript. DJ supervised the research project and provided critical comments. KI, JS, and SK gathered data for the study. All authors contributed to the article and approved the submitted version.

## Funding

The authors acknowledge support from The Maria Grzegorzewska University.

## Conflict of Interest

The authors declare that the research was conducted in the absence of any commercial or financial relationships that could be construed as a potential conflict of interest.

## Publisher’s Note

All claims expressed in this article are solely those of the authors and do not necessarily represent those of their affiliated organizations, or those of the publisher, the editors and the reviewers. Any product that may be evaluated in this article, or claim that may be made by its manufacturer, is not guaranteed or endorsed by the publisher.

## Footnotes

^1^The study was conducted at the beginning of January, taking into consideration that starting to exercise is a popular New Year’s resolution.
